# Aberrant ARMCX1 Expression Is an Independent Predictor of Poor Prognosis in Gastric Cancer

**DOI:** 10.1155/2022/9348917

**Published:** 2022-05-05

**Authors:** Aosi Xie, Puyu Wang, Diqun Chen, Hongxia Zhang

**Affiliations:** ^1^Department of Gastrointestinal Surgery, The First Affiliated Hospital of Shantou University Medical College, Shantou 515043, China; ^2^Department of Obstetrics and Gynecology, The First Affiliated Hospital of Shantou University Medical College, Shantou 515043, China; ^3^Health Care Center, The First Affiliated Hospital of Shantou University Medical College, Shantou 515043, China

## Abstract

ARMCX1 (Armadillo repeat containing X-linked 1) is identified to be the novel tumor suppressor gene related to multiple tumor types. Nonetheless, its effect on gastric cancer (GC) is still poorly understood. The present work determined ARMCX1 level within GC and the relation with clinicopathological characteristics. This work also collected relevant information in The Cancer Genome Atlas (TCGA) database for investigating associations of ARMCX1 with clinicopathologic variables and then validated in our GC cohort. Receiver operating characteristic (ROC) curves were plotted for assessing whether ARMCX1 expression was significant in diagnosing GC. Kaplan-Meier (KM) and Cox regression analyses were conducted for assessing clinicopathological characteristics associated with overall survival (OS) of GC cases. The data from the Human Protein Atlas (HPA) and Gene Expression Omnibus (GEO) databases was also analyzed for further validation, and biological processes (BPs) were identified by gene set enrichment analysis (GSEA). GC tissues showed markedly decreased ARMCX1 level relative to healthy counterparts (*P* < 0.001). Interestingly, ARMCX1 upregulation predicted low differentiation, poor OS, increased invasion, and late tumor stage. In addition, the area under ROC curve (AUC) and *P* value were 0.747 and <0.001, separately. Cases showing ARMCX1 upregulation showed significantly poor prognostic outcome compared with patients showing downregulation (*P* = 0.007). Furthermore, multivariate analysis showed that ARMCX1 upregulation independently predicted the risk of OS (*P* = 0.0017, hazard ratio, 1.089). GSEA analysis identified that several cancer-related pathways, such as focal adhesion, ECM receptor interaction, JAK/STAT, melanoma, WNT, and cancer, were enriched in GCs. We conclude that ARMCX1 serves as the possibly independent biomarker to diagnose and predict GC prognostic outcome.

## 1. Introduction

Gastric cancer (GC) ranks the 5th and 3rd places among all cancers in terms of its morbidity and mortality, with more than a million new GC cases annually [1]. Despite the declining GC morbidity within the last 5 decades, its 5-year overall survival (OS) rate remains low [2]. Gastric cancer shows high aggressiveness, with no typical symptoms; as a result, most GC cases already have advanced diseases or even distant metastasis (DM) at the time of diagnosis [3]. Consequently, it is of urgent need to develop new efficient biomarkers to detect, diagnose, and predict GC prognosis.

Armadillo repeat containing X-linked 1 (ARMCX1), an arm protein lost in epithelial cancer on chromosome X1 (ALEX1), exhibits close localization with additional family members on X chromosome, such as ALEX2 and ALEX3. The encoded ARM (Armadillo) protein family possesses the possible transmembrane domain in N-terminus along with 2 arm repeats related to embryogenesis and tumorigenesis as well as tissue integrity maintenance [4, 5]. ARMCX1 has been shown to participate in cellular activities like growth and apoptosis together with adhesion [6]. Reduced or even undetectable ARMCX1 level is reported within several cancers, like lung cancer (LC), liver cancer, pancreatic cancer, colorectal cancer (CRC), prostate cancer (PCa), and ovarian cancer (OC), and is associated with adverse outcomes [6–8]. Recently, according to one functional work, ARMCX1 upregulation promoted cell apoptosis and suppressed their growth, while ARMCX1 silencing can result in diametrically opposite results [9].

Nonetheless, ARMCX1's clinicopathological and prognosis significance within gastric cancer (GC) is still unknown. Consequently, performing further characterization of ARMCX1 as a reliable biomarker and significant predictor in GC patients is of great importance. Thus, the present work focused on exploring ARMCX1's role in diagnosing and treating GC. We also adopted gene set enrichment analysis (GSEA) to further evaluate potential pathways related to ARMCX1 expression within GC.

## 2. Materials and Methods

We followed those methods of Chen et al. as described below [10].

### 2.1. Sample Acquisition

From 2019 to 2020, this work obtained 52 gastric cancer and 52 corresponding noncarcinoma samples in GC cases at the First Affiliated Hospital of Shantou University Medical College. Thereafter, the surgically collected samples were subject to immediate freezing and preservation under −80°C. The present work gained approval from Institutional Research Ethics Committee of the First Affiliated Hospital of Shantou University Medical College.

### 2.2. Data Collection

We collected clinical information and gene expression profiles in Gene Expression Omnibus (GEO) and TCGA databases. In addition, this work also obtained mRNA and gene expression profiles (*n* = 407, which included 32 healthy controls) and clinical data associated with survival of 435 GC cases (data from TCGA updated to April 7, 2020) in The Cancer Genome Atlas (TCGA) Genomic Data Commons data portal (https://portal.gdc.cancer.gov/repository). Additionally, box and whisker plots were drawn for visually showing discrete variable distribution. Besides, RNA-Seq data from 375 GC cases were also analyzed.

### 2.3. Statistical Analysis

The logistic regression and chi-square test were used for evaluating the association of ARMCX1 level with clinicopathologic parameters. KM and Cox regression were utilized in identifying OS-related clinical factors. Moreover, this work conducted multivariate Cox regression for examining the association of ARMCX1 level with clinicopathological factors, including age, sex, tumor stage, tumor grade, DM, lymph node metastasis (LNM), and invasion depth. The ARMCX1 profiles were classified as low- or high-expression group according to median risk score and expression. SPSS software (V24.0) and R software (V.3.5.1) were used for these statistical analyses.

### 2.4. Gene Set Enrichment Analysis

GSEA represents the computational approach adopted for distinguishing different gene set expression levels between high- and low-expression groups, as well as for exploring pathways and regulatory networks with biological significance. ARMCX1 expression data-related phenotype labels (*n* = 375 cancer tissues) in TCGA database were classified as an ARMCX1-high group or an ARMCX1-low group. Enrichment was defined upon the thresholds of false discovery rate (FDR) *Q* < 0.25 and *P* < 0.05.

### 2.5. Gene Expression Omnibus (GEO) and Human Protein Atlas (HPA) Databases Analysis

For further validating whether conclusions obtained in cohort study using TCGA were accurate, this work also collected GEO dataset for further analyses. GSE26942 included 205 GC as well as 12 adjacent nontumorous tissue samples and were utilized in ARMCX1 differential analysis, whereas GSE15459 involved 192 GC tissues with complete clinical data and was utilized in independently testing ARMCX1 as a prognostic predictor. Meanwhile, the HPA (http://www.proteinatlas.org/) includes the expression map showing the full-length human proteome within cancer and healthy samples. Consequently, HPA-derived immunohistochemistry (IHC) data were analyzed to further validate differential protein levels.

### 2.6. Quantitative Real-Time Polymerase Chain Reaction

This work utilized TRIzol reagent (Thermo Fisher Scientific) for extracting RNA in GC or adjacent samples. Geneseed® II First Strand cDNA Synthesis Kit was utilized to generate cDNA. Primers of cDNA used to amplify ARMCX1 included 5′-GTCG ACGCCACCATGGGCCGC AC-3′ (F); 5 -GTCGACTCAGAGT TTGGTTAAT ACTTTCAGGAC-3′ (R). And the qPCR were performed in a 20 *μ*L reaction volume following specific protocols. The reaction system (20 *μ*L) included 2×qPCR SYBR Green 30 Master Mix (10 *μ*L, Vazyme Biotech), cDNA (5 *μ*L), and respective primers (0.4 *μ*L, 10 *μ*M). Each sample was tested thrice, with GAPDH Being the reference to normalize ARMCX1 mRNA expression.

## 3. Results

### 3.1. Relation of ARMCX1 with Clinical Factors

**Figure 1:**
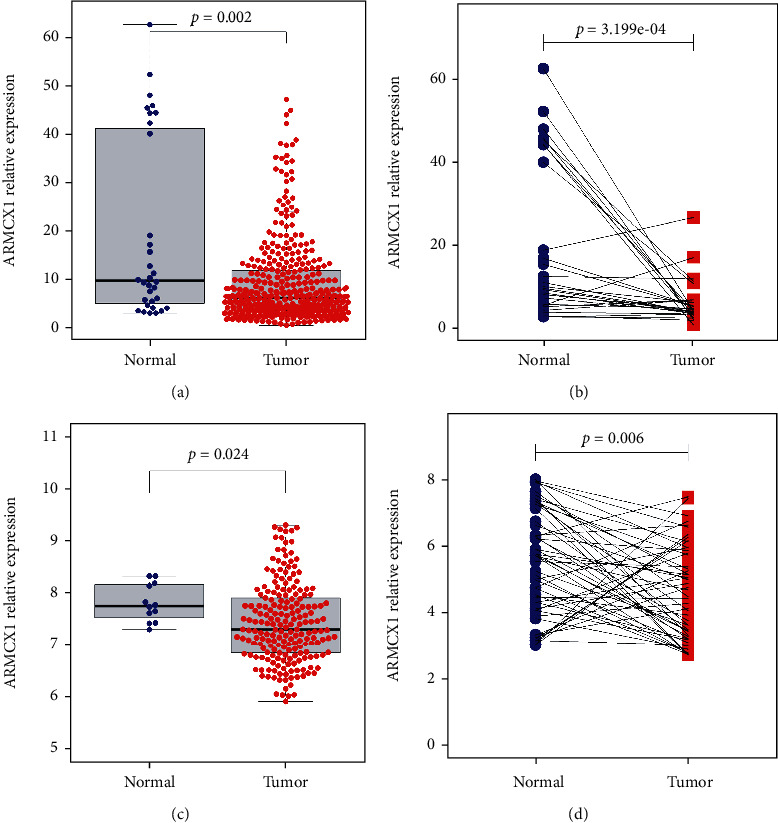
ARMCX1 differential expression in normal and tumor tissues: (a) comparison of group samples in TCGA profiles; (b) comparison of paired samples in TCGA cohort; (c) comparison of group samples in the GEO dataset; (d) comparison of group samples in our validation cohort.

This work identified altogether 407 cases (which included 375 cancer as well as 32 healthy samples) in the GC cohort in TCGA. Relations of ARMCX1 with clinicopathological variables, such as age, sex, tumor stage, tumor grade, invasion depth, DM, and LNM, were analyzed. ARMCX1 level was significantly lower within the cancer samples in comparison with healthy samples (Figure 1(a), *P* = 0.002). Differential analysis within paired samples (GC tissues and normal tissues) showed the same result (Figure 1(b), *P* < 0.001). Different ARMCX1 levels were validated separately using GSE26942 (Figure 1(c), *P* = 0.024) as well as our validation cohort (Figure 1(d), *P* = 0.006).

**Figure 2:**
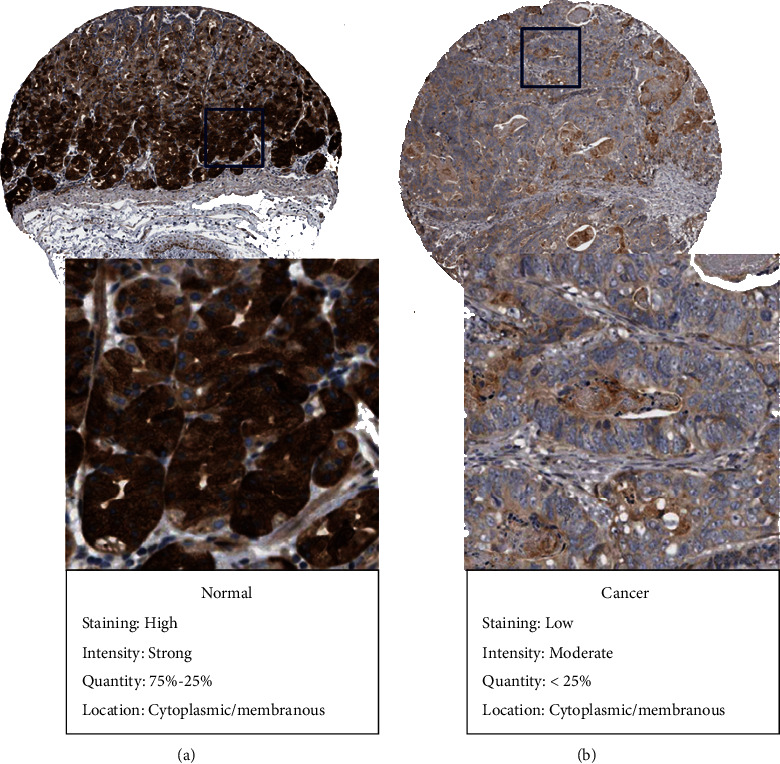
Validation of ARMCX1 protein expression in normal tissue (a) and gastric cancer (b) from the Human Protein Atlas database.

For better validating ARMCX1 protein level, this work visualized IHC staining data in HPA and showed markedly enhanced ARMCX1 staining within glandular cells in healthy stomach samples (Figure 2(a)), whereas gastric cancer tissues had low ARMCX1 staining (Figure 2(b)). These findings confirmed our research results at the mRNA level.

According to Table 1, the ARMCX1 level showed significant relation to age (*P* = 0.019), clinical stage (*P* = 0.041), and tumor grade (*P* = 0.003) as well as local invasion depth (*P* < 0.001) but not to sex, DM, or LNM in the GC cohort in TCGA, confirming the above results and further showing that ARMCX1 expression is correlated with depth of local invasion (*P* = 0.047), TNM stage (*P* = 0.026), and LNM (*P* = 0.026) rather than age, sex, tumor grade, or tumor size. As suggested by univariate logistic regression, ARMCX1 level was related to unfavorable prognostic clinicopathologic factors (Table 2). In TCGA cohort, ARMCX1 upregulation within GC showed significant relation to clinical stage (OR = 2.78 and 2.234 for stages II and III vs. stage I, separately) and T classification (OR = 8.936, 8.50 and 10.818 for T2, T3, and T4 vs. T1, separately). In our validation cohort, high expression of ARMCX1 in GC was markedly related to T classification (OR = 9.048 for T4 vs. T1-T3), TNM stage (OR = 3.600 for stage III vs. stage I&II), and LNM (OR = 3.600 for yes vs. no).

### 3.2. Diagnostic Value of ARMCX1 in GC

**Figure 3:**
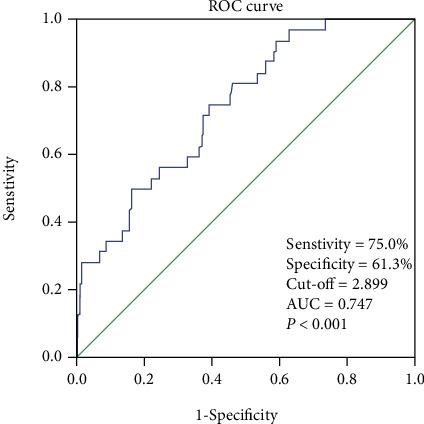
Receiver operating characteristic (ROC) curve for ARMCX1 expression in normal and gastric cancer tissues.

To evaluate the diagnostic value of ARMCX1, the mRNA expression profiles from TCGA (375 GC patients and 32 normal tissues) were assessed by receiver operating characteristic (ROC). The area under the ROC curve was 0.747 [95% confidence interval (CI): 66.6%–82.8%], the sensitivity was 75.0%, and the specificity was 61.3%, which indicates feasible diagnostic value (Figure 3).

### 3.3. Survival Analysis and Univariate/Multivariate Analysis

**Figure 4:**
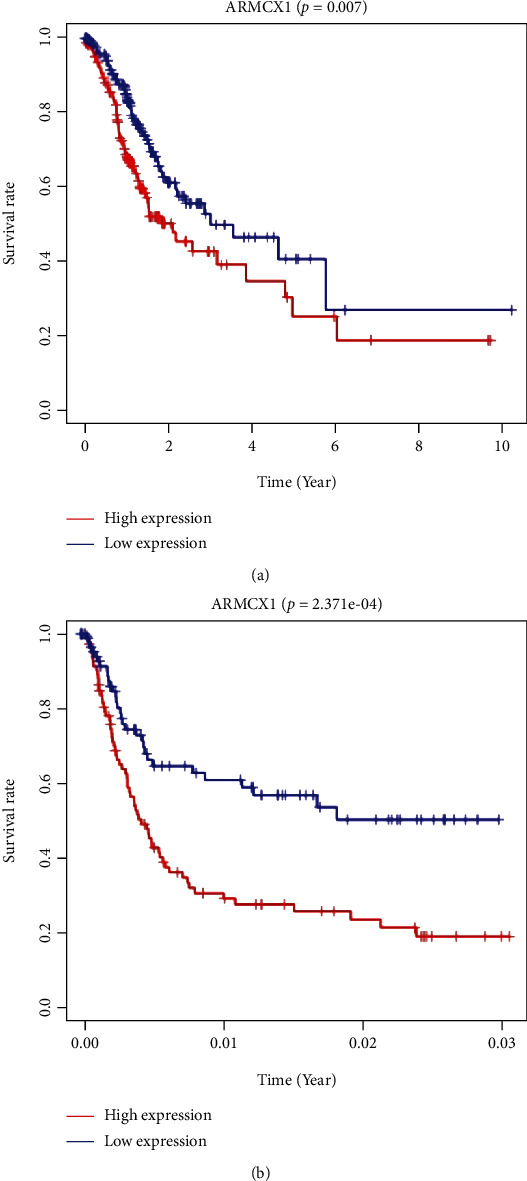
ARMCX1 expression and overall survival in gastric cancer patients in TCGA cohort (a) and the GSE15459 dataset (b).

According to Figure 4, ARMCX1 upregulation showed strong relation to dismal OS (Figure 4(a), *P* = 0.007), and it was confirmed using the GSE15459 cohort (Figure 4(b), *P* < 0.001). According to univariate analysis, overexpression of ARMCX1 predicted the dismal OS [hazard ratio (HR): 1.064; 95% CI: 1.0012–1.118; *P* = 0.0156] (Table 3). In addition, additional factors related to poor OS were age, TNM stage, and stage. Significant clinical variables were incorporated in multivariate Cox regression; as a result, age and ARMCX1 upregulation still independently predicted the OS risk, and the HRs were 1.042 (95% CI: 1.021–1.063, *P* < 0.001) and 1.089 (95% CI: 1.032–1.149, *P* value = 0.0018), respectively. Similarly, upon univariate as well as multivariate analysis based on GSE15459, ARMCX1 upregulation and clinical stage independently predicted the prognosis of poor OS for GC cases (Table 4).

### 3.4. ARMCX1-Related Signaling Pathways by GSEA

**Figure 5:**
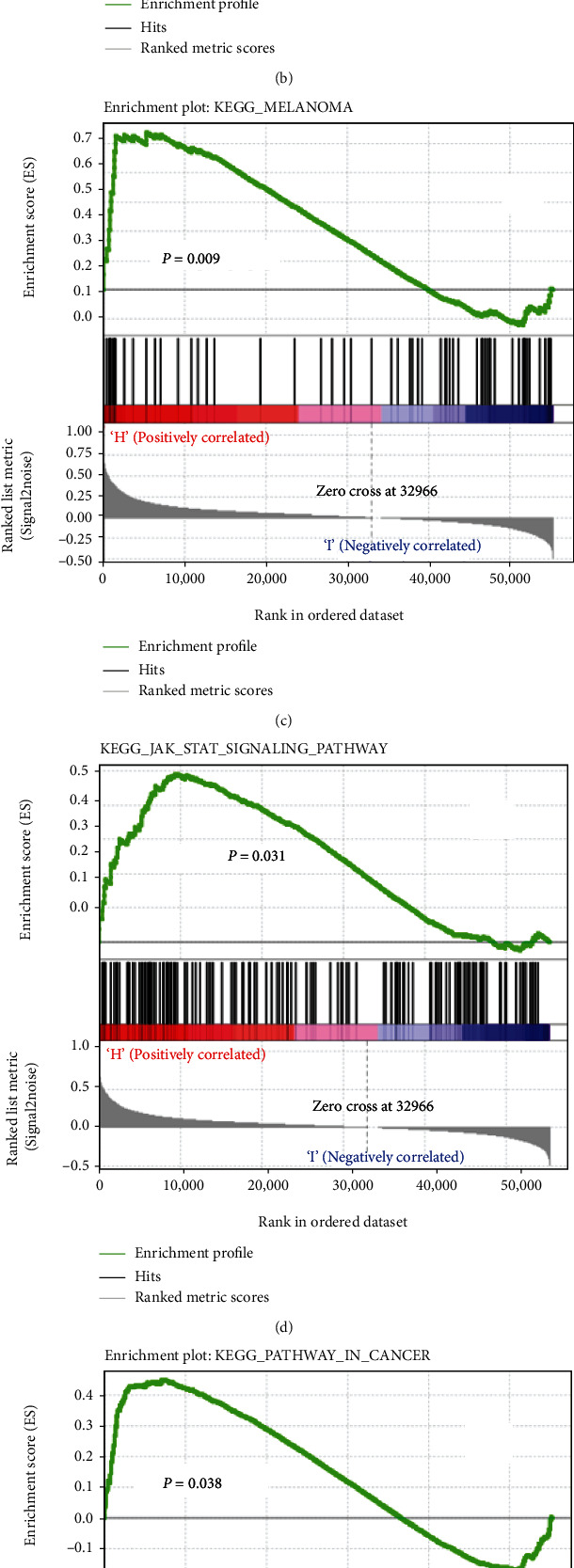
Significantly enriched signaling pathways of GSEA. Genes involved in extracellular matrix-receptor interaction (a), focal adhesion (b), melanoma (c), JAK STAT signaling (d), cancer (e), and WNT signaling (f) were significantly enriched in ARMCX1-related gastric cancer.

This work conducted GSEA for selecting potentially involved pathways through the comparison between low and high ARMCX1 expression groups based on the molecular signatures database (MSigDB). This work performed 1000 random sample permutations. Typically, this work adopted FDR *q* < 0.05 and nominal *P* < 0.05 as the significance thresholds GSEA. According to Figure 5, this work examined multiple cancer-related pathways that were enriched according to enrichment scores normalized, like chemokine signaling, melanoma, extracellular matrix receptor interaction, cancer, Wnt pathway, and Toll-like receptor pathway (FDR < 0.01), and were associated with ARMCX1 upregulation within GC.

## 4. Discussion

ARMCX1 belongs to the armadillo subfamily; it regulates interactions between proteins and is related to transcriptional activation, cell junction assembly, and nuclear transport through interaction with its armadillo repeat domain [4, 11]. Mounting evidence suggests that ARMCX1 plays key roles in embryogenesis and tumorigenesis. For instances, ARMCX1 inhibits colony formation of CRC cells while promoting BC cell apoptosis [6, 9], and ARMCX1 knockdown in immortalized embryonic hepatocytes promotes hepatocarcinogenesis in mice [8]. Importantly, ARMCX1 mRNA is critical downregulated or even undetectable in several carcinomas [7]. Based on these findings, ARMCX1 is the potential tumor suppressor during cancer occurrence and development. But its expression profile as well as the correlation with clinicopathologic factors in GC remains largely unknown.

This work conducted bioinformatics analyses on ARMCX1 expression profiles in GEO and TCGA databases, and ARMCX1 was significantly reduced within cancer tissues in comparison with healthy samples, consistent with a previously published studies [7]. Combined with results from other studies, the ARMCX1 expression level was related to GC's clinicopathological features. According to subsequent analyses, ARMCX1 overexpression was related to age, local invasion depth, clinical stage, and tumor grade. According to one latest work, ARMCX1 level decreased within human GC samples compared to nontumor samples and is correlated with tumor stage and TNM (tumor-node-metastasis) staging [12]. This result conformed to our findings obtained from GC cases.

According to univariate as well as multivariate Cox regression on clinical variables based on GEO and TCGA datasets, ARMCX1 upregulation independently predicted OS. According to ROC curve analysis, ARMCX1 expression had feasible prognostic significance for GC. Moreover, age independently predicted OS of GC, as suggested by multivariate analysis.

For better exploring ARMCX1's effect on GC, this work conducted GSEA for distinguishing key TCGA-derived gene sets. As a result, certain tumor-associated pathways were markedly associated with ARMCX1 upregulation within GC, which included focal adhesion, extracellular matrix receptor interaction, chemokine signaling, cancer, Wnt pathway, and Toll-like receptor pathway.

Recently, extracellular matrix (ECM) is suggested to impact tumor microenvironment (TME), which has a critical effect on cancer development [13]. The ECM has an important effect on cancer cell migration and differentiation, as well as matrix organization via the complicated biological interactions [14, 15]. A recent in silico analyses on differentially expressed genes within GC, changes in cell adhesion, and ECM remodeling cooperate to promote the development or progression of GC [16], which is consistent with our GSEA result. ECM-affecting pathways show interactions with cell adhesion as well, while the imbalanced status of them will lead to cancer development [17].

The Wnt pathway has been extensively identified to be critical for cell growth in the development of cancer and healthy guts [18]. Overexpression of SOX2 (SRY-Box Transcription Factor 2) and SALL4 (Spalt-like Transcription Factor 4) activates the Wnt/*β*-catenin pathway while leading to the worse survival outcome in gastrointestinal cancer [19, 20]. A previous study showed that CRE-binding protein (CREB) upregulation through continuously activating Wnt/beta-catenin pathway increased the ARMCX1 expression within PC and CRC cells, whereas CREB knockdown decreased ARMCX1 expression [21].

According to one recent work, ARMCX1, which directly targets miR-106 in gastric cancer, is upregulated and can rescue the cell apoptosis and promoted the phosphorylation level of JAK-STAT by miR-106b inhibitor, while interestingly, decreased miR-106b expression promotes GC cell apoptosis by suppressing JAK1/STAT3 pathway in vivo and in vitro [22]. These findings might explain why ARMCX1, as a tumor suppressor gene, is overexpressed and related to dismal OS of GC.

## 5. Conclusions

According to GEO and TCGA-based bioinformatics analyses and our experimental validation, the aberrant ARMCX1 level might be the possible biomarker for GC. In addition, extracellular matrix receptor interaction, Wnt signaling, and JAK1/STAT3 signaling pathway may be key pathways of ARMCX1 expression within GC.

## Figures and Tables

**Table 1:** Association between ARMCX1 expression and clinicopathologic characteristics in TCGA and our validation cohort.

TCGA cohort		Total	ARMCX1 expression		*Χ* ^2^	*P*
			Low expression	High expression		
Age	<65	164	71	93	5.504	*0.019*
	≥65	207	115	92		
Gender	Female	134	66	68	0.065	0.799
	Male	241	122	119		
Tumor grade	G1	10	5	5	11.747	*0.003*
	G2	137	85	52		
	G3	219	96	124		
T stage	T1	19	18	1	18.969	*<0.001*
	T2	80	39	41		
	T3	168	89	79		
	T4	100	41	59		
LN metastasis	Yes	246	119	127	2.639	0.104
	No	111	64	47		
Distant metastasis	Yes	25	10	15	1.299	0.254
	No	330	171	159		
TNM stage	Stage I	53	37	16	8.278	*0.041*
	Stage II	111	53	58		
	Stage III	150	74	76		
	Stage IV	38	18	20		
Validation cohort						
Age	<65	28	17	11	2.786	0.095
	≥65	24	9	15		
Gender	Female	16	9	7	0.361	0.548
	Male	36	17	19		
Tumor size	<5 cm	27	12	15	0.693	0.405
	≥5 cm	*25*	14	11		
Tumor grade	G1 + G2	25	12	13	0.077	0.781
	G3	27	14	13		
T stage	T1 + T2 + T3	25	8	15	3.945	*0.047*
	T4	27	19	6		
LN metastasis	Yes	24	16	8	4.952	*0.026*
	No	28	10	18		
TNM stage	Stage I/II	24	16	8	4.952	*0.026*
	Stage III	28	10	18		

Italics indicated *P* < 0.05 demonstrated by the chi-square test. Abbreviations: LN: lymph node metastasis.

**Table 2:** Logistic regression of NUDT10 expression and clinicopathological parameters in TCGA and our validation cohort.

TCGA cohort		TN	OR	95% CI	*P* value
Clinical features					
Age	≥65 vs. <65	371	0.611	0.403-0.922	0.193
Gender	Female vs. male	375	0.946	0.620-1.444	0.799
Grade	G2 vs. G1	146	0.638	0.157-2.332	0.501
	G3 vs. G1	229	0.991	0.269-3.655	0.989
T stage	T2 vs. T1	99	7.216	2.191-32.814	*0.003*∗∗
	T3 vs. T1	187	6.154	1.961-27.163	*0.005*∗∗
	T4 vs. T1	119	24.857	4.839-455.953	*0.002*∗∗
LN metastasis	Yes vs. no	357	0.717	0.456-1.123	0.147
Distant metastasis	Yes vs. no	355	1.866	0.817-4.522	0.148
TNM stage	Stage II vs. stage I	164	2.088	1.076-4.129	*0.031*∗∗∗
	Stage III vs. stage I	203	2.409	1.267-4.706	*0.008*∗∗
	Stage IV vs. stage I	91	2.612	1.120-6.286	*0.028*∗
Validation cohort					
Age	≥65 vs. <65	52	2.576	0.854-8.171	0.098
Gender	Female vs. male	52	1.437	0.441-4.837	0.549
Grade	G3 VS G1 + G2	52	0.858	0.285-2.554	0.781
Tumor size	≥5 cm vs. <5 cm	52	0.629	0.206-1.871	0.406
T stage	T4 VS T1 + T2 + T3	52	9.048	2.718-34.458	*<0.001*∗∗∗
LN metastasis	Yes vs. no	52	3.600	1.173-11.852	*0.029*∗
TNM stage	Stage III vs. stage I/II	52	3.600	1.173-11.852	*0.029*∗

Abbreviations: OR: odds ratio; TN: total number; CI, confidence interval; LN: lymph node. Italics indicate statistical significance of expression level with ∗*P* < 0.05, ∗∗*P* < 0.01, ∗∗∗*P* < 0.001.

**Table 3:** Univariate and multivariate analysis of the correlation between ARMCX1 expression and overall survival in gastric cancer patients.

Parameter	Univariate analysis			Multivariate analysis		
	HR	95% CI	*P* value	HR	95% CI	*P* value
Age	1.027	1.008-1.046	*0.00556*∗	1.042	1.021-1.063	*<0.001*∗
Gender	1.484	0.980-2.247	0.06239	1.662	1.079-2.560	0.0884
Grade	1.368	0.947-1.977	0.09538	1.296	0.876-1.917	0.1946
Stage	1.535	1.221-1.931	*<0.001*∗	1.324	0.856-2.048	0.2072
T	1.298	1.023-1.645	*0.03152*∗	1.132	0.814-1.572	0.4612
M	2.048	1.096-3.827	*0.02458*∗	1.820	0.814-4.072	0.1449
N	1.267	1.069-1.502	*0.00639*∗	1.118	0.871-1.436	0.3817
ARMCX1	1.064	1.012-1.118	*0.01556*∗	1.089	1.032-1.149	*0.0018*∗

∗indicates *P* < 0.05.

**Table 4:** Univariate and multivariate analysis of the correlation between ARMCX1 expression and overall survival in gastric cancer patients in the GSE15459 dataset.

Parameter	Univariate analysis			Multivariate analysis		
	HR	95% CI	*P* value	HR	95% CI	*P* value
Age	1.000	0.984-1.016	0.99839	1.015	0.998-1.031	0.0763
Gender	1.402	0.908-2.166	0.12703	0.778	0.490-1.236	0.2883
Stage	2.787	2.139-3.632	*<0.001*∗	3.019	2.280-3.997	*<0.001*∗
ARMCX1	1.191	1.013-1.400	*0.03403*∗	1.293	1.084-1.541	*0.0042*∗

∗indicates *P* < 0.05.

## Data Availability

The datasets or profiles used in the current study are from TCGA and GEO databases, which are available at the following websites: https://portal.gdc.cancer.gov/repository, https://www.ncbi.nlm.nih.gov/geo/.
